# Supplementary material for: Colorectal cancer-associated PCBP1 mutations disrupt protein stability in a dominant-negative manner
Source: J Biol Chem. 2026 Jul 28;302(9):113376. doi: 10.1016/j.jbc.2026.113376 (PMC13524681; doi:10.1016/j.jbc.2026.113376)
Supplement: Supplementary file 1 [file mmc1.pdf]

SUPPLEMENTARY DATA

Title: Colorectal cancer-associated PCBP1 mutations disrupt protein stability in a dominant negative manner

Author List:

Paige V. Blinkiewicz[1,2], Nicole C. Hoenes[1,2], Myra X. Afzal[3], Yihong Liu[4], Ulysses J. Hill[1,2], Sophie K. Trafton[2], Berglind Bjarnadottir[2], T. Emme Burgin[2,3], Prerna Malaney[1,2,*]

[1]Dartmouth Cancer Center, Lebanon NH
[2]Department of Biochemistry and Cell Biology, Dartmouth College, Hanover NH
[3]Thayer School of Engineering, Dartmouth College, Hanover NH
[4]Department of Biological Sciences, Dartmouth College, Hanover NH

*Corresponding author
Email: prerna.malaney@dartmouth.edu

Supp Fig 1

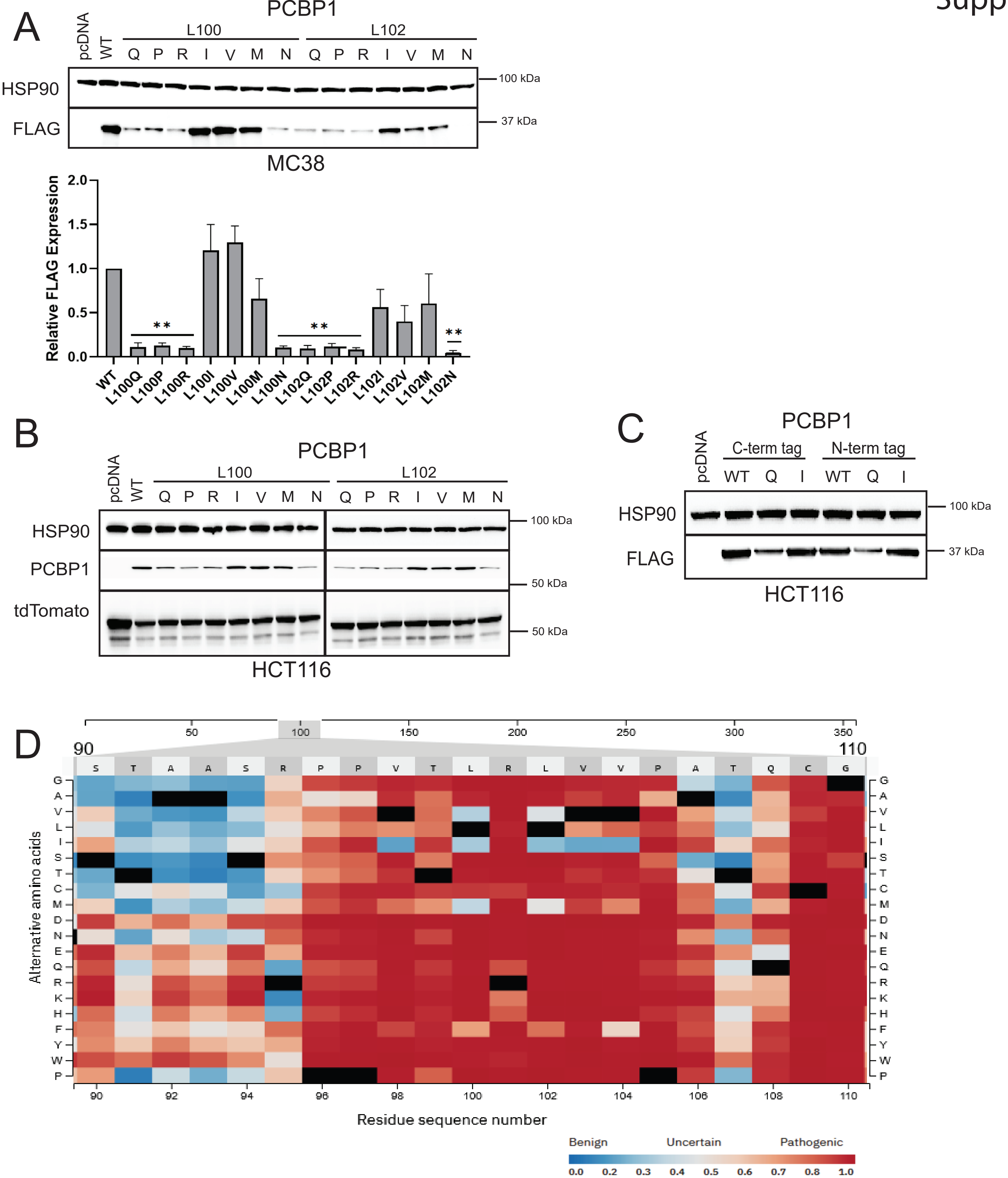
A
PCBP1
pcDNA
WT
L100
L102
Q P R I V M N Q P R I V M N
HSP90
FLAG
100 kDa
37 kDa
MC38
Relative FLAG Expression
2.0
1.5
1.0
0.5
0.0
**
**
**
WT
L100Q
L100P
L100R
L100I
L100V
L100M
L100N
L102Q
L102P
L102R
L102I
L102V
L102M
L102N
B
PCBP1
pcDNA
WT
L100
L102
Q P R I V M N Q P R I V M N
HSP90
PCBP1
tdTomato
100 kDa
50 kDa
50 kDa
HCT116
C
PCBP1
pcDNA
C-term tag
N-term tag
WT Q I WT Q I
HSP90
FLAG
100 kDa
37 kDa
HCT116
D
50
100
150
200
250
300
350
90
110
S T A A S R P P V T L R L V V P A T Q C G
G A V L I S T C M D N E Q R K H F Y W P
Alternative amino acids
90 92 94 96 98 100 102 104 106 108 110
Residue sequence number
Benign
Uncertain
Pathogenic
0.0 0.2 0.3 0.4 0.5 0.6 0.7 0.8 1.0

Supp Fig 1

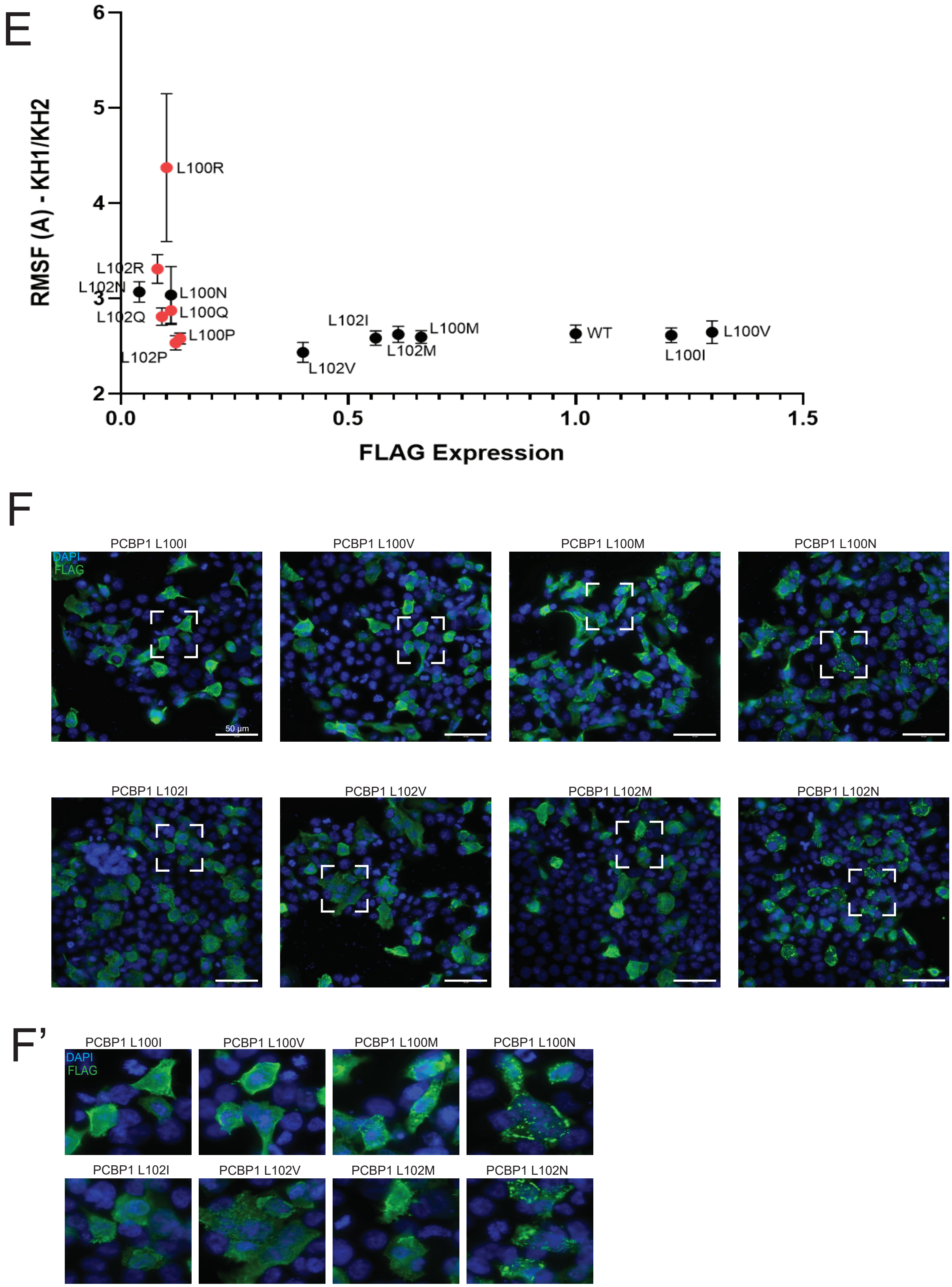
E
RMSF (A) - KH1/KH2
FLAG Expression
6
5
4
3
2
0.0
0.5
1.0
1.5
L100R
L102R
L102N
L100N
L102Q
L100Q
L100P
L102P
L102I
L100M
L102M
L102V
WT
L100I
L100V
F
PCBP1 L100I
PCBP1 L100V
PCBP1 L100M
PCBP1 L100N
DAPI
FLAG
50 µm
PCBP1 L102I
PCBP1 L102V
PCBP1 L102M
PCBP1 L102N
F'
PCBP1 L100I
PCBP1 L100V
PCBP1 L100M
PCBP1 L100N
DAPI
FLAG
PCBP1 L102I
PCBP1 L102V
PCBP1 L102M
PCBP1 L102N

Supplementary Figure 1: Mutations at L100/L102 disrupt PCBP1 protein expression and conformation. (A) Western blot showing expression level for C-terminal Flag-tagged PCBP1 plasmids upon transient transfection in MC38 cells. Quantification of the western blot was done using three independent replicates and p-values were calculated using one-way ANOVA (comparing PCBP1 WT to the various mutants) with a post hoc Dunnett's Test (** $p<0.01$) **(B)** Western blot showing expression for GFP-PCBP1-d2A-tdTomato plasmids upon transient transfection in HCT116 cells. Representative image is shown (n = 3). **(C)** Western blot showing expression level for C-terminal and N-terminal Flag-tagged PCBP1 plasmids upon transient transfection in HCT116 cells. Representative image is shown (n = 3). **(D)** Heat map depicting AlphaMissense data to characterize the effect of different mutations on PCBP1 structure. **(E)** Relative root mean square fluctuation of the KH1-KH2 domains correlated with the expression levels of the various PCBP1 mutants in MC38 cells. RMSF across residues in the KH1 domain are calculated relative to residues in the KH2 domain. **(F and F')** Sub-cellular localization of PCBP1 mutants, as assessed by Flag immunofluorescence, upon transient transfection in HCT116 cells. Representative image is shown (scale = 50 μm; n = 3).

Supp Fig 2

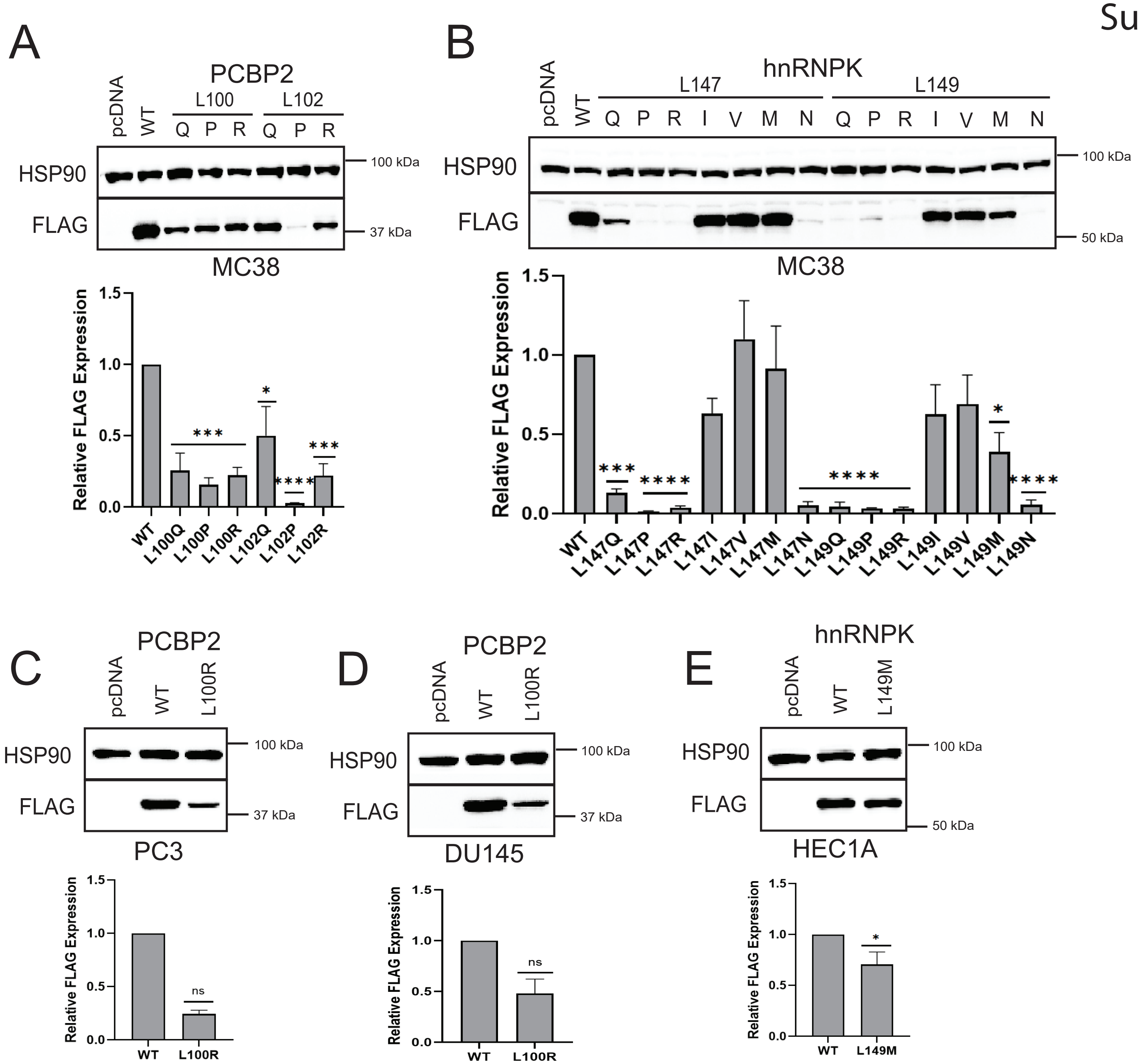
A
PCBP2
pcDNA
WT
L100
L102
Q P R Q P R
HSP90
FLAG
100 kDa
37 kDa
MC38
Relative FLAG Expression
1.5
1.0
0.5
0.0
***
*
****
***
WT L100Q L100P L100R L102Q L102P L102R
B
hnRNPK
pcDNA
WT
L147
L149
Q P R I V M N Q P R I V M N
HSP90
FLAG
100 kDa
50 kDa
MC38
Relative FLAG Expression
1.5
1.0
0.5
0.0
***
****
****
*
****
WT L147Q L147P L147R L147I L147V L147M L147N L149Q L149P L149R L149I L149V L149M L149N
C
PCBP2
pcDNA
WT
L100R
HSP90
FLAG
100 kDa
37 kDa
PC3
Relative FLAG Expression
1.5
1.0
0.5
0.0
ns
WT L100R
D
PCBP2
pcDNA
WT
L100R
HSP90
FLAG
100 kDa
37 kDa
DU145
Relative FLAG Expression
1.5
1.0
0.5
0.0
ns
WT L100R
E
hnRNPK
pcDNA
WT
L149M
HSP90
FLAG
100 kDa
50 kDa
HEC1A
Relative FLAG Expression
1.5
1.0
0.5
0.0
*
WT L149M

Supplementary Figure 2: Mutations at L100/L102 result in reduced expression of other KH domain containing RNA-binding proteins. (A & B) Western blot showing expression level for C-terminal Flag-tagged PCBP2 and Flag-tagged hnRNPK plasmids respectively upon transient transfection in MC38 cells. Quantification of the western blot was done using three independent replicates and p-values were calculated using one-way ANOVA (comparing wild type protein the corresponding mutant proteins) with a post hoc Dunnett's Test (* $p<0.05$; *** $p<0.001$; **** $p<0.0001$). **(C & D)** Western blot showing expression level for prostate cancer-associated C-terminal Flag-tagged PCBP2 L100R mutant in PC3 and DU145 cell lines respectively. Quantification of the western blot was done using three independent replicates and p-values were calculated using Mann Whitney test (ns $p>0.05$). **(E)** Western blot showing expression level for endometrial cancer-associated C-terminal Flag-tagged hnRNPK L149M mutant in HEC1A cells. Quantification of the western blot was done using three independent replicates and p-values were calculated using Mann Whitney test (* $p<0.05$).

Supp Fig 3

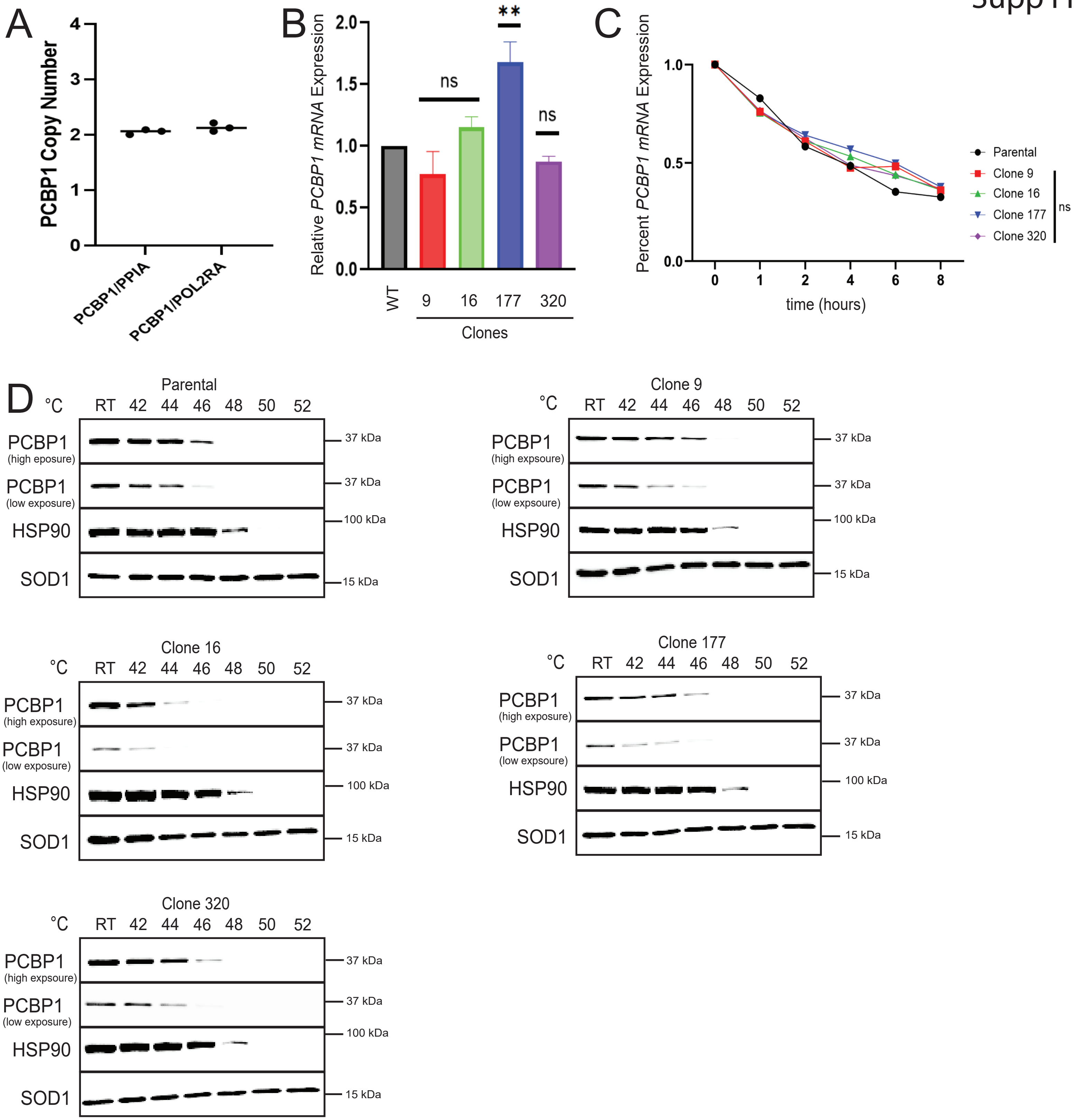

A
PCBP1 Copy Number
PCBP1/PPIA
PCBP1/POL2RA
B
Relative PCBP1 mRNA Expression
ns
**
ns
WT
9
16
177
320
Clones
C
Percent PCBP1 mRNA Expression
time (hours)
Parental
Clone 9
Clone 16
Clone 177
Clone 320
ns
D
Parental
°C
RT 42 44 46 48 50 52
PCBP1 (high eposure)
PCBP1 (low exposure)
HSP90
SOD1
37 kDa
37 kDa
100 kDa
15 kDa
Clone 9
PCBP1 (high expsoure)
PCBP1 (low expsoure)
Clone 16
PCBP1 (high exposure)
PCBP1 (low exposure)
Clone 177
PCBP1 (high exposure)
PCBP1 (low expsoure)
Clone 320
PCBP1 (high expsoure)
PCBP1 (low exposure)

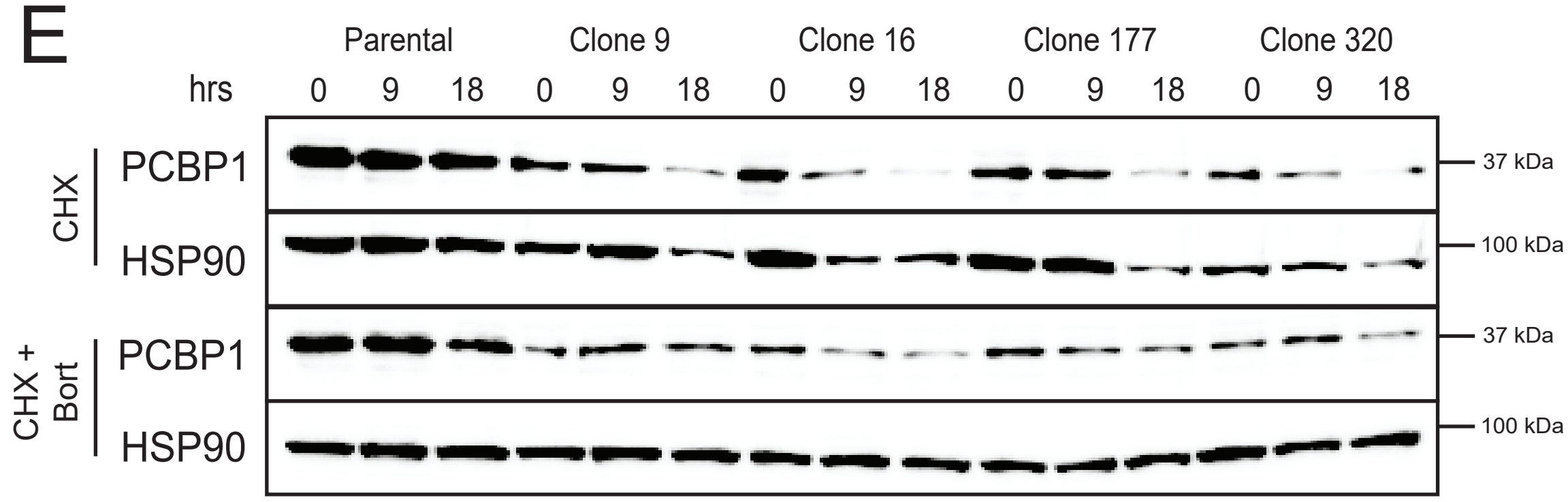
E
Parental
Clone 9
Clone 16
Clone 177
Clone 320
hrs
0 9 18 0 9 18 0 9 18 0 9 18 0 9 18
CHX
PCBP1
HSP90
CHX + Bort
PCBP1
HSP90
37 kDa
100 kDa
37 kDa
100 kDa

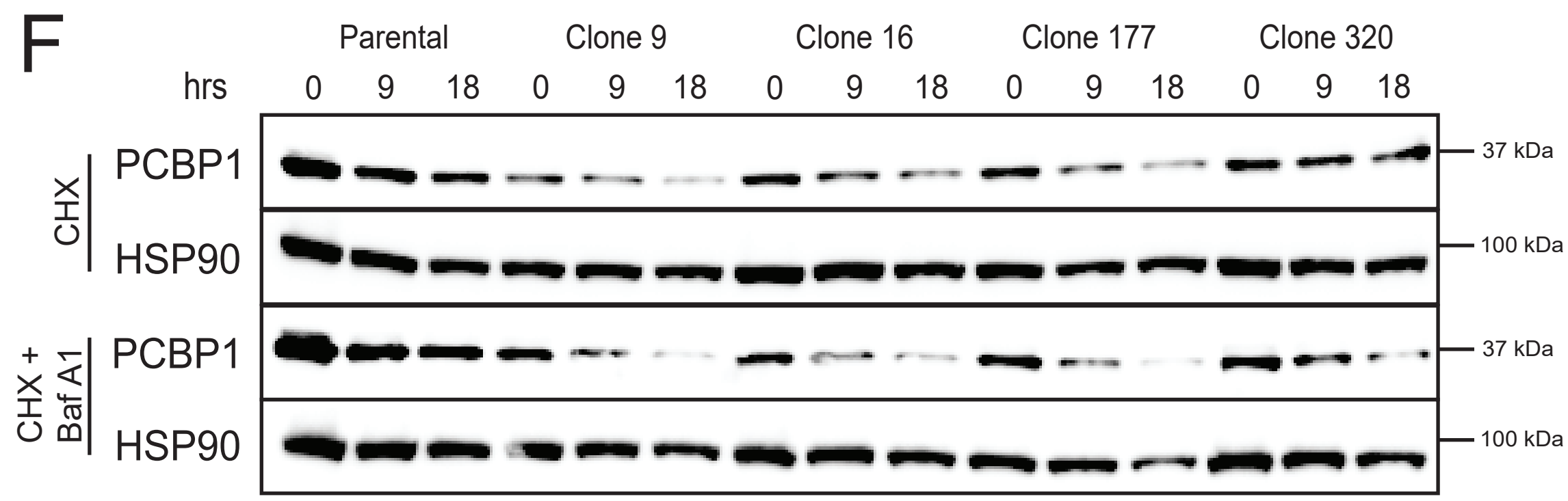
F
Parental
Clone 9
Clone 16
Clone 177
Clone 320
hrs
0 9 18 0 9 18 0 9 18 0 9 18 0 9 18
CHX
PCBP1
HSP90
CHX + Baf A1
PCBP1
HSP90
37 kDa
100 kDa
37 kDa
100 kDa

Supplementary Figure 3: The L100Q mutation destabilizes PCBP1 protein resulting in increased total PCBP1 turnover in PCBP1$^{L100Q/WT}$ cell lines. (A) Copy number analysis for PCBP1 in HCT116 cells as assessed by droplet digital PCR. **(B)** *PCBP1* transcript levels in HCT116 PCBP1$^{WT/WT}$ and HCT116 PCBP1$^{L100Q/WT}$ cells as determined by qPCR. *PPIA* was used as a housekeeping control. Quantification was performed on three biological replicates and p-values were calculated using one-way ANOVA with a post hoc Dunnett's Test (** $p<0.01$; ns $p>0.05$). **(C)** Actinomycin chase assay to determine *PCBP1* mRNA stability in HCT116 PCBP1$^{WT/WT}$ and HCT116 PCBP1$^{L100Q/WT}$ cell lines. Quantification was performed on three biological replicates and p-values were calculated using two-way ANOVA with a post hoc Dunnett's Test (ns $p>0.05$). **(D)** Cellular thermal shift assay to determine the melting temperature of total PCBP1 in HCT116 PCBP1$^{WT/WT}$ parental cells and HCT116 PCBP1$^{L100Q/WT}$ mutant clones. HSP90: positive control; SOD1: negative control. Representative image is shown (n = 3). **(E)** Cycloheximide chase assays with and without proteasomal inhibitor, bortezomib, in HCT116 PCBP1$^{WT/WT}$ and HCT116 PCBP1$^{L100Q/WT}$ cells. Representative image is shown (n = 2). **(F)** Cycloheximide chase assays with and without lysosomal inhibitor, bafilomycin A1, in HCT116 PCBP1$^{WT/WT}$ and HCT116 PCBP1$^{L100Q/WT}$ cells. Representative image is shown (n = 2).

Supp Fig 4

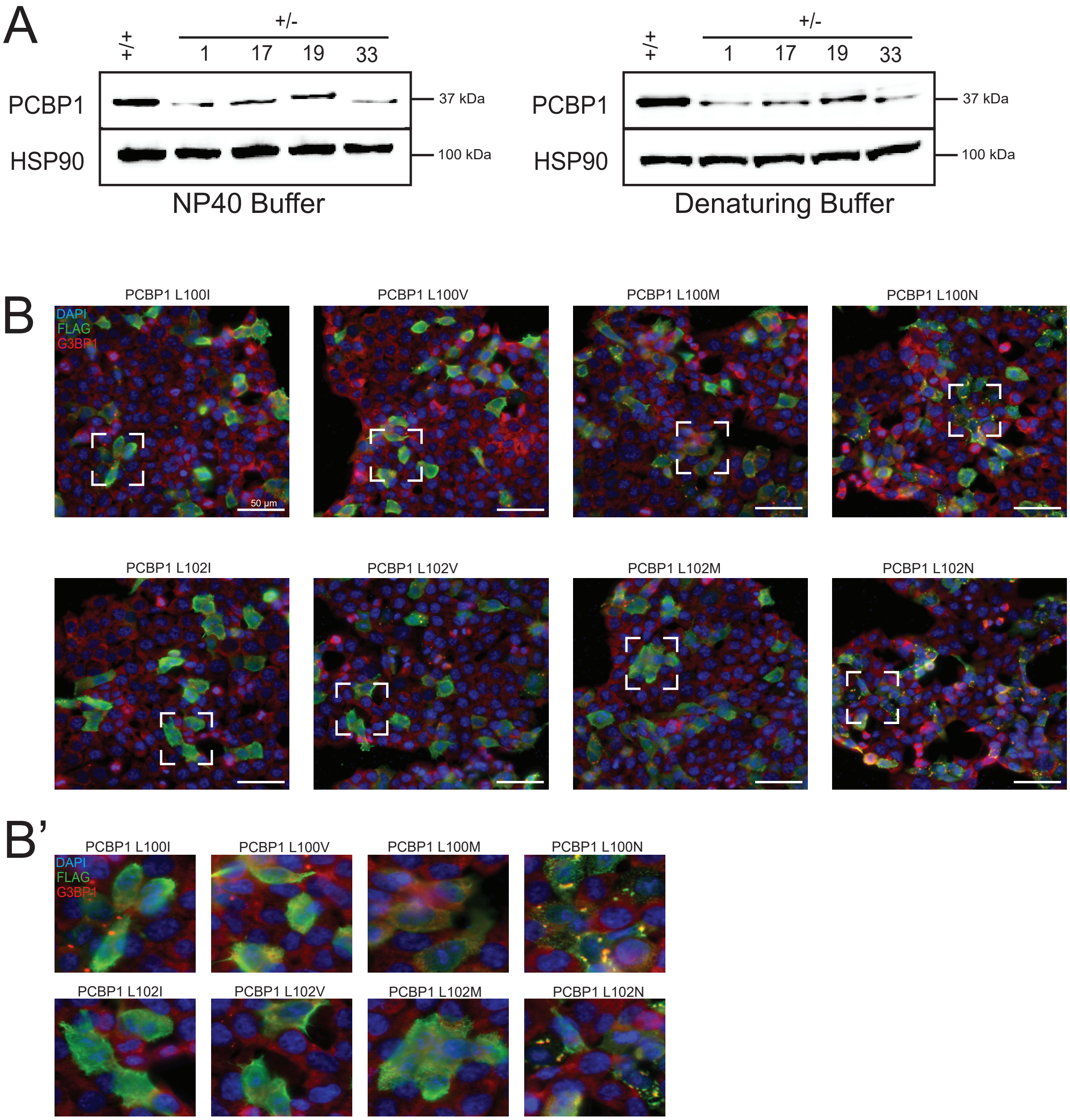
A
+/+
+/-
1
17
19
33
PCBP1
HSP90
37 kDa
100 kDa
NP40 Buffer
Denaturing Buffer
B
PCBP1 L100I
PCBP1 L100V
PCBP1 L100M
PCBP1 L100N
DAPI
FLAG
G3BP1
50 µm
PCBP1 L102I
PCBP1 L102V
PCBP1 L102M
PCBP1 L102N
B’
PCBP1 L100I
PCBP1 L100V
PCBP1 L100M
PCBP1 L100N
DAPI
FLAG
G3BP1
PCBP1 L102I
PCBP1 L102V
PCBP1 L102M
PCBP1 L102N

Supp Fig 4

C

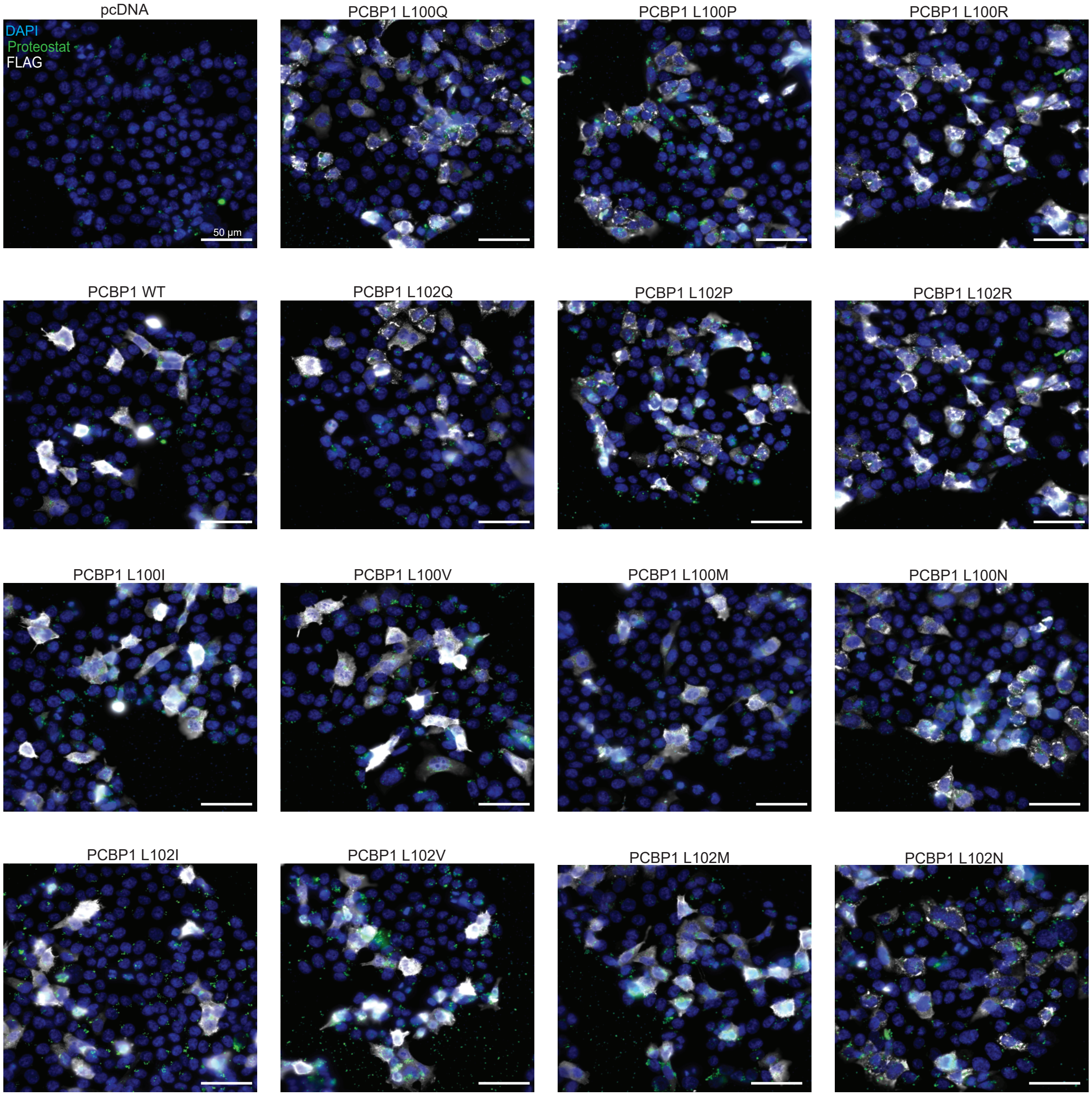

pcDNA
DAPI
Proteostat
FLAG
50 µm
PCBP1 L100Q
PCBP1 L100P
PCBP1 L100R
PCBP1 WT
PCBP1 L102Q
PCBP1 L102P
PCBP1 L102R
PCBP1 L100I
PCBP1 L100V
PCBP1 L100M
PCBP1 L100N
PCBP1 L102I
PCBP1 L102V
PCBP1 L102M
PCBP1 L102N

Supplementary Figure 4: PCBP1 L100/L102 mutant proteins are insoluble. (A) Western blot showing expression level for total PCBP1 in HCT116 PCBP1$^{+/+}$ and HCT116 PCBP1$^{+/-}$ cells lysed in NP40 or denaturing buffer. Representative image is shown (n = 3). **(B & B')** Co-localization of Flag-PCBP1 mutants, as assessed by Flag and G3BP1 immunofluorescence, upon transient transfection in HCT116 cells. Representative image is shown (scale = 50 µm; n = 3). **(C)** Co-localization of Flag-PCBP1 mutants, as assessed by Flag and proteostat immunofluorescence, upon transient transfection in HCT116 cells. Representative image is shown (scale = 50 µm; n = 2).

Supp Fig 5

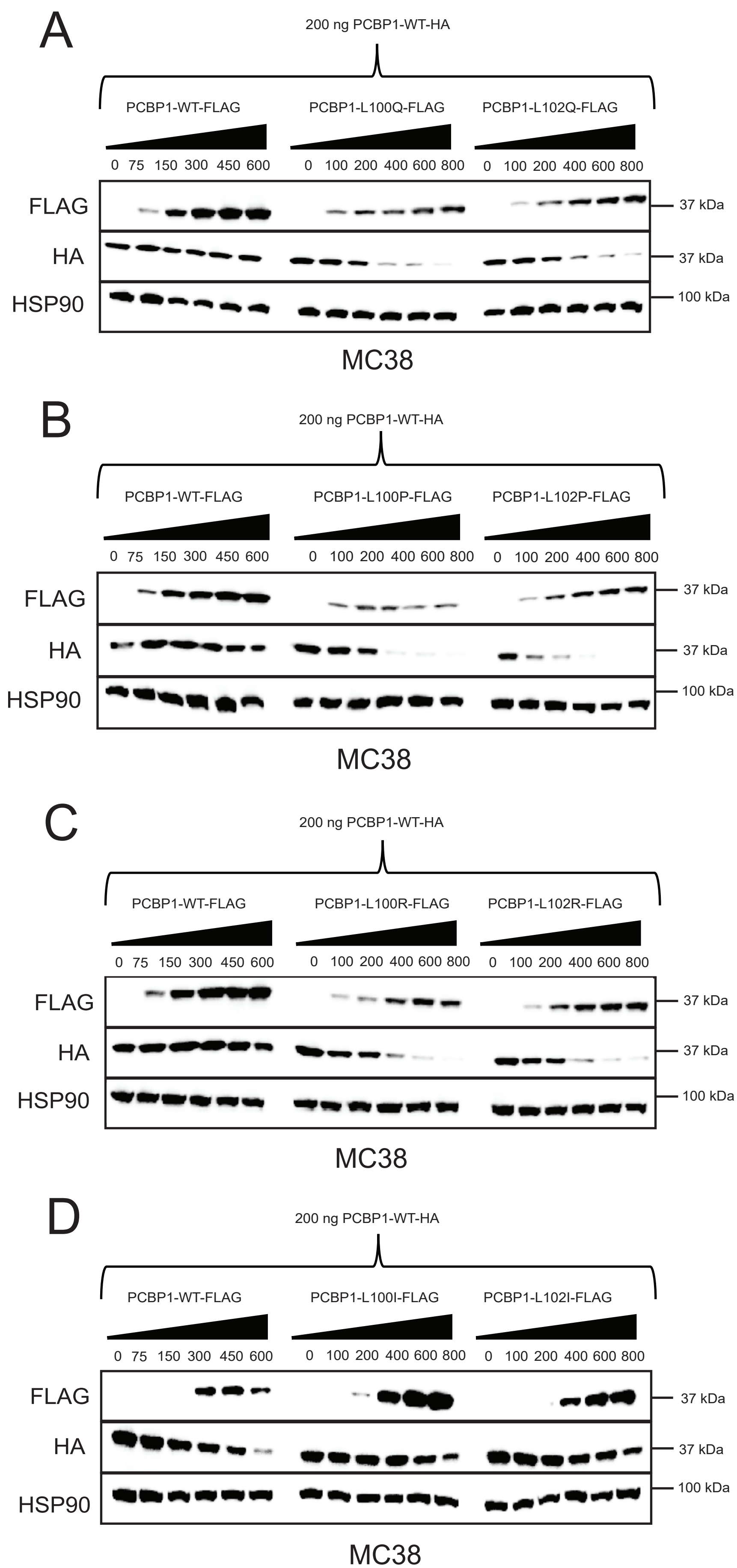
A
200 ng PCBP1-WT-HA
PCBP1-WT-FLAG
PCBP1-L100Q-FLAG
PCBP1-L102Q-FLAG
0 75 150 300 450 600
0 100 200 400 600 800
0 100 200 400 600 800
FLAG
HA
HSP90
37 kDa
37 kDa
100 kDa
MC38
B
200 ng PCBP1-WT-HA
PCBP1-WT-FLAG
PCBP1-L100P-FLAG
PCBP1-L102P-FLAG
0 75 150 300 450 600
0 100 200 400 600 800
0 100 200 400 600 800
FLAG
HA
HSP90
37 kDa
37 kDa
100 kDa
MC38
C
200 ng PCBP1-WT-HA
PCBP1-WT-FLAG
PCBP1-L100R-FLAG
PCBP1-L102R-FLAG
0 75 150 300 450 600
0 100 200 400 600 800
0 100 200 400 600 800
FLAG
HA
HSP90
37 kDa
37 kDa
100 kDa
MC38
D
200 ng PCBP1-WT-HA
PCBP1-WT-FLAG
PCBP1-L100I-FLAG
PCBP1-L102I-FLAG
0 75 150 300 450 600
0 100 200 400 600 800
0 100 200 400 600 800
FLAG
HA
HSP90
37 kDa
37 kDa
100 kDa
MC38

Supp Fig 5

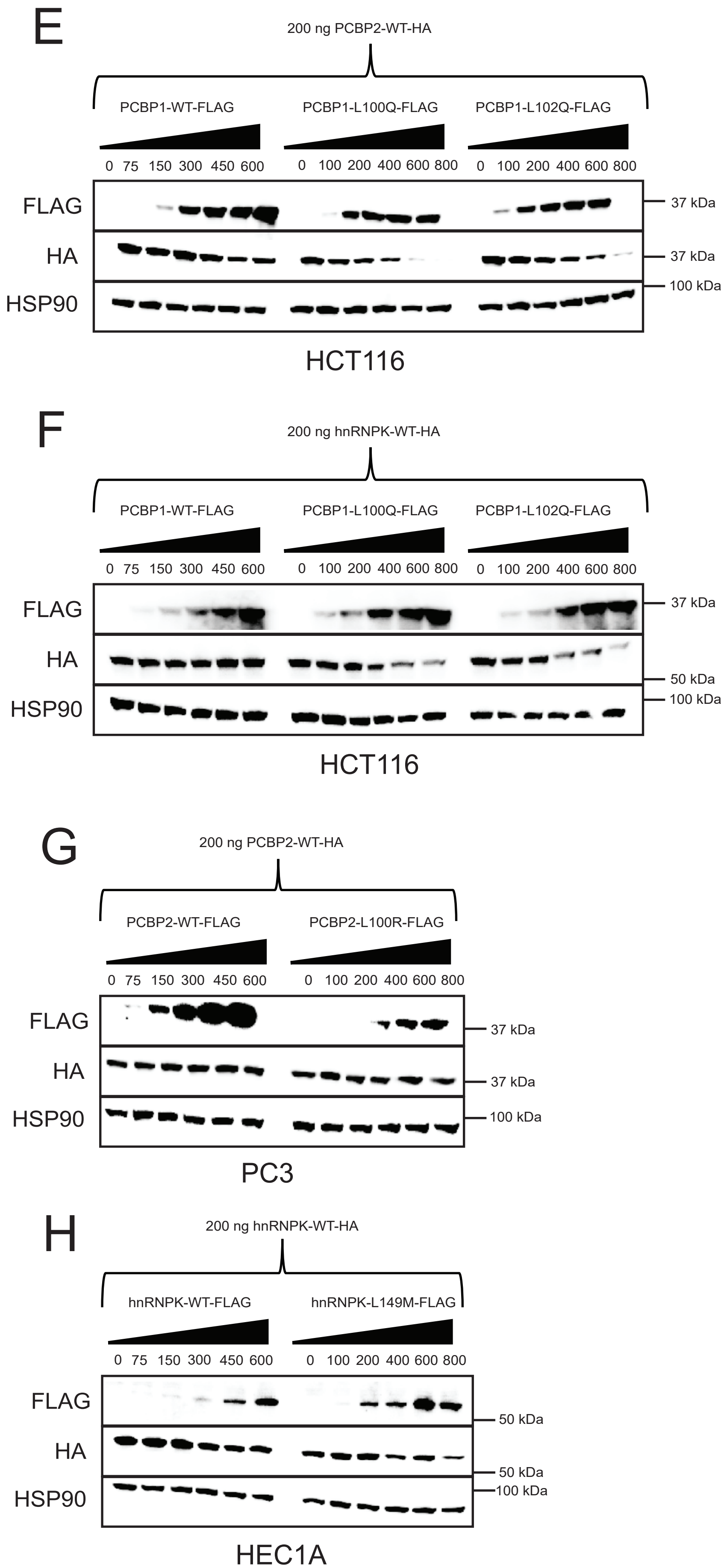
E
200 ng PCBP2-WT-HA
PCBP1-WT-FLAG
PCBP1-L100Q-FLAG
PCBP1-L102Q-FLAG
0 75 150 300 450 600
0 100 200 400 600 800
0 100 200 400 600 800
FLAG
HA
HSP90
37 kDa
37 kDa
100 kDa
HCT116
F
200 ng hnRNPK-WT-HA
PCBP1-WT-FLAG
PCBP1-L100Q-FLAG
PCBP1-L102Q-FLAG
0 75 150 300 450 600
0 100 200 400 600 800
0 100 200 400 600 800
FLAG
HA
HSP90
37 kDa
50 kDa
100 kDa
HCT116
G
200 ng PCBP2-WT-HA
PCBP2-WT-FLAG
PCBP2-L100R-FLAG
0 75 150 300 450 600
0 100 200 400 600 800
FLAG
HA
HSP90
37 kDa
37 kDa
100 kDa
PC3
H
200 ng hnRNPK-WT-HA
hnRNPK-WT-FLAG
hnRNPK-L149M-FLAG
0 75 150 300 450 600
0 100 200 400 600 800
FLAG
HA
HSP90
50 kDa
50 kDa
100 kDa
HEC1A

Supplementary Figure 5: PCBP1 L100/L102 mutants act in a dominant negative manner to suppress wild type PCBP1 expression. (A-D) Western blots to assess the dominant negative nature of the PCBP1 mutants. MC38 cells were transiently transfected with a combination of 200 ng of HA-tagged PCBP1 WT and the indicated amounts of the Flag-tagged PCBP1 mutants. Representative image is shown (n = 3). **(E-F)** Western blots to assess the dominant negative nature of the PCBP1 mutants on wild type PCBP2 and hnRNPK expression levels. HCT116 cells were transiently transfected with a combination of 200 ng of HA-tagged PCBP2 WT (E) or HA-tagged hnRNPK WT (F) and the indicated amounts of the Flag-tagged PCBP1 mutants. Representative image is shown (n = 3). **(G)** Western blots to assess the dominant negative nature of the PCBP2 L100R mutant. PC3 cells were transiently transfected with a combination of 200 ng of HA-tagged PCBP2 WT and the indicated amounts of the Flag-tagged PCBP2 mutants. Representative image is shown (n = 3). **(H)** Western blots to assess the dominant negative nature of the hnRNPK L149M mutant. HEC1A cells were transiently transfected with a combination of 200 ng of HA-tagged hnRNPK WT and the indicated amounts of the Flag-tagged hnRNPK mutants. Representative image is shown (n = 3).

Supplementary Tables

Supplementary Table 1: List of primers used in this study

**Primer Name**	**Sequence**
PCBP1 FL-F	ATGCCTCGAGCTCGCCATGGATGCCGGTGTGACTGAAA
PCBP1 FL-Flag-R	ATGCGAATTCCTACTTGTCATCGTCGTCCTTGTAGTCGCTGCACCCCATGCCCTT
PCBP1 FL-HA-R	ATGCGAATTCCTAAGCGTAATCTGGAACATCGTATGGGTAGCTGCACCCCATGCCCTT
PCBP1 L100Q-F	GGTCACCCAGAGGCTGGTG
PCBP1 L100Q-R	CACCAGCCTCTGGGTGACC
PCBP1 L100P-F	GGTCACCCCGAGGCTGGTG
PCBP1 L100P-R	CACCAGCCTCGGGGTGACC
PCBP1 L100R-F	GGTCACCCGGAGGCTGGTG
PCBP1 L100R-R	CACCAGCCTCCGGGTGACC
PCBP1 L100I-F	GGTCACCATCAGGCTGGTG
PCBP1 L100I-R	CACCAGCCTGATGGTGACC
PCBP1 L100V-F	GGTCACCGTGAGGCTGGTG
PCBP1 L100V-R	CACCAGCCTCACGGTGACC
PCBP1 L100M-F	GGTCACCATGAGGCTGGTG
PCBP1 L100M-R	CACCAGCCTCATGGTGACC
PCBP1 L100N-F	GGTCACCAACAGGCTGGTG
PCBP1 L100N-R	CACCAGCCTGTTGGTGACC
PCBP1 L102Q-F	CCCTGAGGCAGGTGGTGC
PCBP1 L102Q-R	GCACCACCTGCCTCAGGG
PCBP1 L102P-F	CACCCTGAGGCCGGTGGT
PCBP1 L102P-R	ACCACCGGCCTCAGGGTG
PCBP1 L102R-F	CACCCTGAGGCGGGTGGT
PCBP1 L102R-R	ACCACCCGCCTCAGGGTG
PCBP1 L102I-F	CCCTGAGGATCGTGGTGC
PCBP1 L102I-R	GCACCACGATCCTCAGGG
PCBP1 L102V-F	CCCTGAGGGTGGTGGTGC
PCBP1 L102V-R	GCACCACCACCCTCAGGG
PCBP1 L102M-F	CCCTGAGGATGGTGGTGC
PCBP1 L102M-R	GCACCACCATCCTCAGGG
PCBP1 L102N-F	CCCTGAGGAACGTGGTGC
GFP-d2A-tdTomato PCBP1-F	ATGCGGTACCATGGATGCCGGTGTGACTGAAA
GFP-d2A-tdTomato PCBP1-R	ATGCGGTACCGCTGCACCCCATGCCCTT

PCBP1 L102N-R	GCACCACGTTCCTCAGGG
NFlag-PCBP1-F	ATGCCTCGAGCTCGCCATGGACTACAAGGACGACGATGACAAGGATGCCGGTGTGACTGAAA
NFlag-PCBP1-R	ATGCGAATTCCTAGCTGCACCCCATGCC
hnRNPK FL-F	ATGCCTCGAGATAAAAGAATATGGAAACTGAACAGCCAGAAGAAAC
hnRNPK FL-Flag-R	ATGCGAATTCTTACTTGTCATCGTCGTCCTTGTAGTCGAAAAACTTTCCAGAATACTGCTTCACACT
hnRNPK FL-HA-R	ATGCGAATTCTTAAGCGTAATCTGGAACATCGTATGGGTAGAAAAACTTTCCAGAATACTGCTTCACACT
hnRNPK L147Q-F	GACTGCGAGCAGAGGCTGTTG
hnRNPK L147Q-R	CAACAGCCTCTGCTCGCAGTC
hnRNPK L147P-F	CTGCGAGCCGAGGCTGTT
hnRNPK L147P-R	AACAGCCTCGGCTCGCAG
hnRNPK L147R-F	CTGCGAGCGGAGGCTGTT
hnRNPK L147R-R	AACAGCCTCCGCTCGCAG
hnRNPK L147I-F	GACTGCGAGATCAGGCTGTTG
hnRNPK L147I-R	CAACAGCCTGATCTCGCAGTC
hnRNPK L147V-F	GACTGCGAGGTGAGGCTGTTG
hnRNPK L147V-R	CAACAGCCTCACCTCGCAGTC
hnRNPK L147M-F	GACTGCGAGATGAGGCTGTTG
hnRNPK L147M-R	CAACAGCCTCATCTCGCAGTC
hnRNPK L147N-F	GACTGCGAGAACAGGCTGTTG
hnRNPK L147N-R	CAACAGCCTGTTCTCGCAGTC
hnRNPK L149Q-F	GAGTTGAGGCAGTTGATTCATCAG
hnRNPK L149Q-R	CTGATGAATCAACTGCCTCAACTC
hnRNPK L149P-F	TTGAGGCCGTTGATTCATCAG
hnRNPK L149P-R	CTGATGAATCAACGGCCTCAA
hnRNPK L149R-F	TTGAGGCGGTTGATTCATCAG
hnRNPK L149R-R	CTGATGAATCAACCGCCTCAA
hnRNPK L149I-F	TGCGAGTTGAGGATCTTGATTCATCAG
hnRNPK L149I-R	CTGATGAATCAAGATCCTCAACTCGCA
hnRNPK L149V-F	TGCGAGTTGAGGGTGTTGATTCATCAG
hnRNPK L149V-R	CTGATGAATCAACACCCTCAACTCGCA
hnRNPK L149M-F	TGCGAGTTGAGGATGTTGATTCATCAG
hnRNPK L149M-R	CTGATGAATCAACATCCTCAACTCGCA
hnRNPK L149N-F	TGCGAGTTGAGGAACTTGATTCATCAG
hnRNPK L149N-R	CTGATGAATCAAGTTCCTCAACTCGCA
PCBP2 FL - F	ATGCCTCGAGCTGCTCGACATGGACACCGGTGTGATTGAAGGT
PCBP2 FL FLAG – R	ATGCGAATTCCTACTTGTCATCGTCGTCCTTGTAGTCGCTGCTCCCCATGCCACC

PCBP2 FL HA - R	ATGCGAATTCCTAAGCGTAATCTGGAACATCGTATGGGTAGCT GCTCCCCATGCCACC
PCBP2 L100Q - F	CCGGTCACCCAGAGGCTGGTG
PCBP2 L100Q - R	CACCAGCCTCTGGGTGACCGG
PCBP2 L100P - F	CCGGTCACCCCGAGGCTG
PCBP2 L100P - R	CAGCCTCGGGGTGACCGG
PCBP2 L100R - F	CCGGTCACCCGGAGGCTG
PCBP2 L100R - R	CAGCCTCCGGGTGACCGG
PCBP2 L102Q - F	ACCCTGAGGCAGGTGGTC
PCBP2 L102Q - R	GACCACCTGCCTCAGGGT
PCBP2 L102P - F	ACCCTGAGGCCGGTGGTC
PCBP2 L102P - R	GACCACCGGCCTCAGGGT
PCBP2 L102R - F	ACCCTGAGGCGGGTGGTC
PCBP2 L102R - R	GACCACCCGCCTCAGGGT
PCBP1-F	CCTGACCAACAGTACCGC
PCBP1-R	TACACCCGCCTTTCCCAATC
PPIA-F	CCCACCGTGTTCTTCGACATT
PPIA-R	GGACCCGTATGCTTTAGGATGA

Supplementary Table 2: List of antibodies used in this study

Antibody	Supplier	Catalog Number	Experiment	Dilution
Anti-Flag M2 produced in mouse	Sigma	F1804-200UG	WB, IF	1:1000
Anti-Flag produced in rabbit	Sigma	F7425-.2MG	WB	1:1000
HA-Tag	Cell Signaling Technology	3724	WB	1:1000
Anti-mCherry	Novus	NBP1-96752	WB	1:2000
Anti-G3BP1	Proteintech	13057-2-AP	IF	1:1000
Anti-PCBP1	Abcam	AB168377	WB	1:1000
Anti-PCBP1	Thermofisher	PA5-65369	IF	1:200
Anti-HSP90	Enzo	ADI-SPA-836-F	WB	1:5000
Anti-SOD1	Cell Signaling Technlogy	2770S	WB	1:1000
Mouse anti-rabbit IgG	Jackson Immuno Research	211-032-171	WB	1:10,000
Goat anti-mouse IgG	Jackson Immuno Research	115-035-174	WB	1:10,000
Invitrogen™ Donkey anti-Mouse IgG (H+L) Highly Cross-Adsorbed Secondary Antibody, Alexa Fluor™ 488	Thermo Fisher Scientific	A21202	IF	1:1000
Invitrogen™ Goat anti-Mouse IgG (H+L) Highly Cross-Adsorbed Secondary Antibody, Alexa Fluor™ Plus 594	Thermo Fisher Scientific	PIA32742	IF	1:1000
Invitrogen™ Goat anti-Mouse IgG (H+L) Highly Cross-Adsorbed Secondary Antibody, Alexa Fluor™ Plus 647	Thermo Fisher Scientific	PIA32728TR	IF	1:1000
Invitrogen™ Goat anti-Rabbit IgG (H+L) Highly Cross-Adsorbed Secondary Antibody, Alexa Fluor™ Plus 488	Thermo Fisher Scientific	PIA32731	IF	1:1000
Invitrogen™ Goat anti-Rabbit IgG (H+L) Highly Cross-Adsorbed Secondary Antibody, Alexa Fluor™ 594	Thermo Fisher Scientific	A11037	IF	1:1000
Invitrogen™ Goat anti-Rabbit IgG (H+L) Highly Cross-Adsorbed	Thermo Fisher Scientific	PIA32733	IF	1:1000

Secondary Antibody, Alexa Fluor™ Plus 647				
DAPI	Sigma	D9542	IF	1:2000
